# Histamine Receptor 3 negatively regulates oligodendrocyte differentiation and remyelination

**DOI:** 10.1371/journal.pone.0189380

**Published:** 2017-12-18

**Authors:** Yongfeng Chen, Wei Zhen, Tony Guo, Yonggang Zhao, Ailian Liu, Justin P. Rubio, David Krull, Jill C. Richardson, Hongtao Lu, Ryan Wang

**Affiliations:** 1 Neuro-immunology Discovery Performance Unit, GSK, Shanghai, China; 2 RD Platform Technology & Science, GSK, Shanghai, China; 3 Genetics, Projects Clinical Platforms & Sciences, GSK, Stevenage, Herts, United Kingdom; 4 Pathology, RD Platform Technology & Science, GSK, Research Triangle Park, NC, United States of America; 5 Neuroinflammation DPU, Neurosciences TAU, GSK, Stevenage, Herts, United Kingdom; Instituto Cajal-CSIC, SPAIN

## Abstract

**Background:**

Agents promoting oligodendrocyte precursor cell differentiation have the potential to restore halted and/or delayed remyelination in patients with multiple sclerosis. However, few therapeutic targets have been identified. The objective of this study was to identify novel targets for promotion of remyelination and characterize their activity *in vitro* and *in vivo*.

**Methods:**

A high-content screening assay with differentiation of primary rat oligodendrocyte precursor cells was used to screen GSK-proprietary annotated libraries for remyelination-promoting compounds. Compounds were further validated *in vitro* and *in vivo* models; clinical relevance of target was confirmed in human post-mortem brain sections from patients with MS.

**Results:**

Of ~1000 compounds screened, 36 promoted oligodendrocyte precursor cell differentiation in a concentration-dependent manner; seven were histamine receptor-3 (H3R) antagonists. Inverse agonists of H3R but not neutral antagonists promoted oligodendrocyte precursor cell (OPC) differentiation. H3R was expressed throughout OPC differentiation; H3R expression was transiently upregulated on Days 3–5 and subsequently downregulated. H3R gene knockdown in OPCs increased the expression of differentiation markers and the number of mature oligodendrocytes. Overexpression of full-length H3R reduced differentiation marker expression and the number of mature cells. H3R inverse agonist GSK247246 reduced intracellular cyclic AMP (cAMP) and downstream cAMP response element-binding protein (CREB) phosphorylation in a dose-dependent manner. Histone deacetylase (HDAC-1) and Hes-5 were identified as key downstream targets of H3R during OPC differentiation. In the mouse cuprizone/rapamycin model of demyelination, systemic administration of brain-penetrable GSK247246 enhanced remyelination and subsequently protected axons. Finally, we detected high H3R expression in oligodendroglial cells from demyelination lesions in human samples of patients with MS, and validated a genetic association between an exonic single nucleotide polymorphism in *HRH3* and susceptibility to multiple sclerosis.

**Conclusions:**

From phenotypic screening to human genetics, we provide evidence for H3R as a novel therapeutic target to promote remyelination in patients with multiple sclerosis.

## Introduction

Multiple sclerosis (MS) is a chronic inflammatory demyelinating disease of the central nervous system (CNS) affecting over 2.3 million people worldwide [1]. MS pathology involves both cellular and humoral autoimmune attacks that primarily target myelin in the CNS, leading to demyelination, gliosis and axonal degeneration [2]. Patients with MS have markedly impaired myelin tissue repair [3–5], which has profound consequences for axon integrity, the progressive and irreversible loss of which accounts for disease progression [6].

It is now widely recognized that remyelination in the CNS may be a highly effective regenerative process to maintain integrity of demyelinated axons [6–8]. Therefore, a new treatment paradigm that restores myelin repair is urgently required to complement current immunomodulatory treatments that only target the inflammatory component of MS and, to date, have not addressed the progression of the disease.

Remyelination is mediated by oligodendrocyte precursor cells (OPCs), and one underlying reason for the impaired myelin tissue repair seen in patients with MS is failure of adult OPCs to differentiate into myelinating oligodendrocytes [3–5]. Although various transcription factors, cell surface receptors and ligands have been shown to regulate oligodendrocyte differentiation and maturation, relatively little is known about the signaling mechanisms that mediate the transition from OPCs to mature oligodendrocytes, and very few drug targets for myelin repair have been identified through hypothesis-driven research. Thus we established a phenotypic screening assay to identify novel remyelination targets, the results of which suggested histamine receptor 3 (H3R) as a potential target for remyelination.

H3R, is a G_i/o_ protein-coupled receptor, believed to regulate release of neurotransmitters in the CNS [9], and has attracted interest as a potential drug target for the treatment of various chronic neurological disorders, including Alzheimer’s disease and schizophrenia [10–13]. Although previously associated with oligodendrocytes, studies to date have focused on its role in neuronal function, and its expression profile and function in the oligodendrocyte lineage remains unclear [10].

This study aimed to investigate the role of H3R in oligodendrocyte differentiation, and to validate and characterize the activity of compounds with the ability to promote *in vitro* OPC differentiation and *in vivo* remyelination.

## Materials and methods

### Phenotypic screening and investigation of OPC differentiation

#### OPC isolation

Enriched OPCs were isolated from 2-day-old rat forebrains as described by Chen *et al*. 2007 [14]. Re-triturated cortex cells were re-suspended with Dulbecco's Modified Eagle Medium (DMEM) supplemented with fetal bovine serum (20%; 10099, Gibco), L-glutamine (50x; 35050, Gibco^TM^), sodium pyruvate (100x; 11360, Gibco) and penicillin/streptomycin (100x; 15140, Invitrogen), and plated onto poly-D-lysine-coated (100 μg/ml; P0899, Sigma-Aldrich®) flasks. After 2 weeks of culture, mixed glial were shaken for 1 hour at 100 rpm to remove microglia and for 20–22 hours at 37°C at 200 rpm to enrich OPCs. Following centrifugation (1200 rpm for 5 minutes) the OPC pellet was re-suspended in basal chemically-defined medium (BDM), which consisted of DMEM (11960, Sigma), N2 (110x; 17502048, Invitrogen), L-glutamine (50x; 35050, Gibco), sodium pyruvate (100x; 11360, Gibco), penicillin/streptomycin (100x; 15140, Invitrogen), bovine serum albumin (0.1%; A9647, Sigma), D-biotin (10 nM; B4501, Sigma), and hydrocortisone (10 nM; H0888, Sigma). OPCs were plated on poly-ornithine (PO)-coated (50 μg/ml; P2533, Sigma) plates and maintained in BDM supplemented with basic fibroblast growth factor (bFGF) (10 ng/ml; PHG0263, Invitrogen) and platelet-derived growth factor (PDGF 10 ng/ml; 100-13A, Peprotech). OPCs were 95% pure judged by A2B5+ staining.

#### OPC differentiation assay and data analysis

Prior to differentiation, selected compounds from GSK-proprietary annotated libraries (single dose, 1 μM; full dose curve, 0.3 nM–10 μM, duplicate) were added into the PO-coated 384-miniwell plates by an Echo machine (Thermo). Cultured OPCs were digested with Trypsin/ ethylenediaminetetraacetic acid (EDTA, 0.05%; 25200, Invitrogen) and seeded onto compound-loaded plates at a density of 2000 cells/well. OPCs were cultured in BDM supplemented with N-acetyl-L-cysteine (30 μM; A-8199, Sigma), PDGF and bFGF (1 ng/ml, each) for 96 hours to differentiate into mature oligodendrocytes. The resulting cultures were fixed with paraformaldehyde (4%; 15812, Sigma), incubated at 4°C overnight in primary antibody (anti-myelin basic protein [MBP] antibody) or negative/positive controls (dimethyl sulfoxide [DMSO] or Triiodothyronine [T3]), washed thoroughly, and incubated with Alexa 488-labeled secondary antibody (Molecular Probes) for 1.5 hours and 4′,6-diamidino-2-phenylindole (DAPI) (D9542, Sigma) for 0.5 hours at room temperature.

The full-plate immunocytochemistry staining was read by an automatic image-based analysis system–Cellomics (Thermo) and the Cellomics Target Activation bioapplication, and the raw data for each well was acquired to determine the percentage of MBP-positive cells. Data are presented as fold-change of the vehicle well. Full dose curves were generated by Microsoft Excel and IDBS XL fit5 and curve fitting was performed by a four-Parameter Logistic Model embedded in the software (dose response one site, f (x) 201: fit = (A+((B-A)/(1+((x/C)^D)))) using software XL fit5 to derive the half maximal effective concentration (EC_50_) values.

Based on a three-fold increase in MBP staining induced by T3 (15 nM) compared with DMSO, compounds that had the ability to increase the percentage of MBP-positive cells by more than three times that of the standard deviation, compared with the mean of the vehicle group, were selected for further testing.

### H3R expression and downstream signaling activity

H3R expression and its downstream signaling cascade were further validated in a series of experiments using pharmacological tools or gene manipulation. Commercial H3R tool compounds were from Tocris; GSK compounds, such as GSK247246, were from GSK compound management. Compounds were dissolved in DMSO (100%) and then diluted into a series of concentrations; the final concentration of DMSO in *in vitro* assays was 0.1%.

Rabbit anti-MBP (1:800; AB980, Millipore), mouse anti- myelin-associated glycoprotein (MAG) (1:1000; Ab89780, Abcam), mouse anti-O4 (1:500; Mab345, Millipore), rabbit anti-H3R (1:500; Ab15860, Millipore), rabbit anti-H3R (1:100; AB9189, Chemicon), goat anti-PDGFRα (1:100; AF1062, R&D), glutathione S-transferase (GST)-π (1:200; ADI-MSA-102, ENZO), were used for immunostaining experiments. Mouse anti-β-Actin (1:3000; A3854, Sigma-Aldrich), rabbit anti-Hes-5 (1:500; ab107593, Abcam), mouse anti-p-CREB (1:500; 9196, Cell Signaling Technology), and rabbit anti-t-CREB (1:500; 9197, Cell Signaling Technology) antibodies were used as primary antibodies to perform western blots.

#### Western blot

Protein was extracted using radio immunoprecipitation assay (RIPA) lysis buffer (10x; 9806, Cell Signaling Technology) and 10–15 μg of protein was electrophoresed using bis-tris polyacrylamide gels (4–12%; NP0336, ThermoFisher Scientific). Target proteins were transferred to nitrocellulose membranes which were probed with the relevant antibodies. Signal was detected by SuperSignal West Femto Maximum Sensitivity Substrate (34096, Thermo).

#### Immunofluorescent staining of OPCs

OPC cultures were fixed with 4% paraformaldehyde. Fixed cells were incubated in primary antibody overnight at 4°C, washed thoroughly and incubated with Alexa 488-labeled secondary antibody (1:800; Molecular Probes) for 2 hours at room temperature.

#### OPC differentiation and H3R expression in other cell populations

To determine H3R expression at different stages of OPC differentiation, OPCs were cultured in BDM supplemented with N-acetyl-L-cysteine (30 μM) to differentiate into mature oligodendrocytes. Cells were extracted for western blotting on Days 0, 3, 5, and 8. Microglia and astrocytes were also isolated from the mixed glial culture of cortex, as described by Skaper *et al*. 2012 [15]. Schwann cells were isolated from 4-day-old rat sciatic nerve, as described by Tao 2013 [16].

#### OPC transfection

OPCs were transfected with small interfering RNA (siRNA) or plasmid DNA, as previously described by Dugas *et al*. 2006 [17]. Cell pellets of 2–3x10^6^ OPCs were resuspended in 100 μl of nucleofection reagent (VPG-1009; Amaxa, Lonza) containing either 100 pmol of SMARTpool rat Hrh3 siRNA (L-093904-01, Dharmacon), 100 pmol SMARTpool rat CREB1 siRNA (L-092995-00, Dharmacon), 100 pmol of si-control non-targeting small interfering RNA (siRNA) pool (D-001810-10, Dharmacon), or H3R-overexpression plasmid or vector control (both from GSK internal bio-reagent bank) and electroporated (Amaxa nucleofection apparatus, program O-17). Transfected OPCs were cultured on PO-coated plates for 4 days in BDM supplemented with N-acetyl-L-cysteine (30 μM) before western blot or immunofluorescence analysis.

#### H3R expression in OPCs in corpus callosum of naïve mouse brain

Immunostaining was carried out in snap-frozen brain sections from naïve mice. Antigen retrieval (1:10; S1699, DakoCytomation) was performed before blocking sections with blocking buffer (3% normal donkey serum). Following incubation with rabbit anti-H3R/goat anti-PDGFRα, primary antibodies were detected with a combination of Alexa 488 donkey anti-rabbit immunoglobulin G (IgG 1:500; A21206, Invitrogen) and Alexa 594 donkey anti-goat IgG (1:100; A11058, Invitrogen). Images were taken using a confocal microscope (Nikon A1R).

#### cAMP assay

OPCs were treated with a range of GSK247246 doses (30 nM, 100 nM, 300 nM, 1 μM, 3 μM and 10 μM) or DMSO for 30 minutes, and then treated with the same doses of GSK247246 or DMSO plus forskolin (3 μM; F6886, Sigma) for 15 minutes. The cAMP assay was carried out according to the cAMP chemiluminescent immunoassay kit (T1502, Applied Biosystems) manufacturer’s instructions and read using a SpectraMax®M5 Reader (Molecular Devices).

#### CREB phosphorylation measurement

OPCs were treated with a range of GSK247246 doses (100 nM, 300 nM, and 1 μM) or DMSO for 30 minutes. Prior to harvesting, OPCs were treated with forskolin (100 nM) for 30 minutes) as a positive control.

#### Quantitative polymerase chain reaction (Q-PCR)

Total RNA was isolated using an RNeasy Mini Kit (Qiagen) and first-strand cDNA was synthesized using a Sensiscript RT Kit (Qiagen) according to the manufacturer’s instructions. mRNA expression was determined by RT-PCR using SYBR Green Master mix under standard thermocycler conditions (Applied Biosystems). Data were collected and quantitatively analyzed on an ABI Prism 7900 sequence detection system (Applied Biosystems). The cycle threshold (Ct) of each well was used for analysis. ΔCt was obtained using the Ct of Hes5 minus corresponding Ct of actin. ΔΔCt was obtained using ΔCt minus the ΔCt of the Day 1 control. The fold of control was generated as 2^(-ΔΔCt) and subjected to statistical analysis. Sequences of PCR primer pairs were: β-actin (internal control): forward 5'-GCGTCCACCCGCGAGTACAAC-3’, reverse 5'-CGACGACGAGCGCAGCGATA-3'; Hrh3: forward 5′- TACTGTGTGCCTCCTCGGTCTT-3′, reverse 5′- AGCTCGAGTGACTGACAGGAATC-3′; Hes-5: forward 5’- CCGCATCAACAGCATT-3’, reverse 5’- GGTGCCGCGCAACT-3’.

#### Adenovirus transfection

To assess the effect of Hes-5 overexpression, OPCs were plated at a final density of 1x10^6^ cells/well in a 6-well plate in BDM supplemented with N-acetyl-L-cysteine (30 μM). After 1 day of culture, OPCs were transfected with pHBAd-MCMV-Hes-5 and pHBAd-MCMV-GFP (both purchased from Hanbio) adenovirus, and treated with GSK247246 (300 nM), or DMSO as control. OPCs were cultured for a further 24–48 hours before harvest.

#### H3R expression in MS demyelinating lesions

To determine the H3R expression profile in oligodendrocytes in MS white matter lesions, immunostaining was carried out on snap-frozen post-mortem brain sections from patients with MS, obtained from the Netherlands Brain Bank, using both anti-H3R and anti-Neurite Outgrowth Inhibitor A (NogoA) antibodies. Immunostaining with anti-MBP was also performed to distinguish regions of central lesion, lesion edge and non-lesion, and Nogo-A was used as a lineage marker to confirm oligodendrocyte H3R expression.

Paraffin sections were dewaxed and placed in Diva antigen retrieval solution (Biocare Medical) and incubated at 96°C for 30 minutes in the Decloaking Chamber (Biocare Medical). Slides were stained using the Intellipath FLX stainer (Biocare Medical) and sections were blocked in Background Sniper (Biocare Medical). Following incubation of rabbit anti-H3R/ sheep anti-Nogo A/ mouse anti-MBP, primary antibodies were detected with a combination of 594 donkey anti-rabbit IgG (A21207, Invitrogen)/488 donkey anti-goat IgG (A11055, Invitrogen)/donkey anti-sheep Alexa 647/donkey anti-mouse Alexa 488/donkey anti-rabbit Alexa 555. Sections were counterstained with DAPI.

Regions of interest on fluorescently labeled sections were manually drawn on the scanned images to measure expression outside of lesion, along lesion margins and within lesions. Lesions were identified based on loss of MBP staining. Expression of each marker was measured based on fluorescent intensity for each of the 4 channels (DAPI+ Nuclei– 405 violet laser, Alexa 488 MBP– 488 blue argon laser, Alexa 555 H3R – 532 green diode laser and Alexa 647 Nogo A– 633 HeNe laser). Voltage settings and offset values for each photo multiplier tube (PMT) were empirically set to optimize signal:noise ratio. High-resolution field scans were collected within each region and signal intensity was measured for each pixel in each channel.

The chromogenic immunohistochemistry method was developed on the Leica Bond-Max immunostainer and epitope retrieval was performed using Bond ER2 reagent (both Leica Biosystems). Endogenous peroxidase was blocked in hydrogen peroxide solution (3%). H3R antibody (LifeSpan Bio, Biorbyt, US Biological Life Sciences, Abcam, or Novus Biologicals) was detected using the Bond Refine anti-rabbit horseradish peroxidase (HRP) polymer. Counterstaining involved 3,3'-diaminobenzidine (DAB) chromogen, Bond DAB enhancer and hematoxylin. Co-localization of the Novus H3R with antibodies against Nogo A (AF3515; R&D Systems) or oligo2 was performed using a sequential HRP detection protocol followed by Bond Refine alkaline phosphatase and fast red chromogen detection.

### GSK247246 activity in mouse models of demyelination

#### Animals

Male C57BL/6J mice were obtained from the breeding colony of the Shanghai Sippr-BK Laboratory Animal Co. Ltd, and all studies were conducted in accordance with the GSK Policy on the Care, Welfare and Treatment of Laboratory Animals and were reviewed and approved by Institutional Animal Care and Use Committee (IACUC) of GSK R&D China. Mice were housed five per cage with food and water available ad libitum, and were kept on a daily 12-hour light-dark cycle. Temperature and humidity were controlled.

#### Cuprizone model

The cuprizone mouse model was treated according to a modified version of the Armstrong *et al*. 2002 protocol [18]. Briefly, 8-week-old C57/BI6 mice were fed with 0.2% (w/w) cuprizone (bis[cyclohexanone]oxaldihydrazone; Sigma-Aldrich) for 5 weeks to induce demyelination, followed by a 9-day recovery period during which cuprizone was removed from the diet and mice were administered with vehicle control or GSK247246 twice daily. GSK247246 was formulated as a suspension using 1% aqueous methylcellulose as the vehicle.

#### Cuprizone/rapamycin model

The cuprizone/rapamycin mouse model was treated according to the Dutta *et al*. 2013 protocol [19]. Briefly, 8-week old C57/Bl6 mice were fed with 0.2% (w/w) cuprizone and received a daily injection of intraperitoneal rapamycin (10 mg/kg body weight) for 5 weeks, following which cuprizone and rapamycin were withdrawn and mice were treated with GSK247246 (30 mg/kg) twice daily for an additional 9 days prior to sacrifice. Rapamycin solution was prepared by dilution in PEG-400 (5%), Tween 80 (5%), and ethanol (4%).

#### Myelin staining and quantification

Dissected mouse brains were post-fixed in paraformaldehyde (4%) overnight then incubated in 30% sucrose for 24–48 hours. After frozen embedding with isopentane and OCT, whole brain 30 μm coronal sections were taken using a cryostat (MICROM HM525) from -2.46–1.18 mm relative to the bregma [20]. Black-Gold II staining was performed according to a protocol adapted from the manufacturer (AG105, Millipore). Briefly, two free-floating forebrain sections were stained with Black-Gold II solution (0.3%) at 60°C for 20 minutes. Stained slides were scanned by ScanScope (Aperio Technologies Inc) and digital images were captured using ImageScope at a magnification of 20x. Image-Pro 6.3 software (Media Cybernetics, Bethesda, MD, USA) was used for subsequent quantitative evaluation. Baseline settings were set to Black-Gold II staining for each corpus callosum image and maintained during processing. Mean density was calculated as: integrated optical density/total area and the result was expressed as percentage of mean density of naïve control. Data from two sections of forebrain from each animal were analyzed and group data are expressed as mean ± standard error of the mean. Graphs were generated by GraphPad5 PRISM software (GraphPad Software, Inco, San Diego, CA, USA).

#### Immunofluorescence staining

For immunofluorescence staining, 30 μm frozen brain coronal brain sections were placed in DakoCytomation (S1699, Dako) antigen retrieval solution and incubated at 85°C for 20 minutes in 2 ml tubes. Following incubation of goat anti-PDGFRα (1:100; AF1062, R&D Systems) or rabbit anti-GST-π (1:200; ADI-MSA-102, Enzo) antibodies, primary antibodies were detected with Alexa 594 donkey anti-rabbit IgG (1:500; A21207, Invitrogen) or Alexa 488 donkey anti-goat IgG (1:500; A11055, Invitrogen). Sections were counterstained with DAPI (Sigma).

For image analysis of GST-π-positive or PDGFRα-positive cells, three areas of central corpus callosum per section were captured by confocal microscope (Nikon A1R). For each area, the forebrain corpus callosum was outlined and quantified using Nikon confocal software. GST-π-positive or PDGFRα-positive cells within these areas were counted either manually (for GST-π-positive cells) or using Image-Pro 6.3 software (Media Cybernetics, Bethesda, MD, USA) (for PDGFRα-positive cells) in a blinded manner by an independent person. The total number of GST-π-positive or PDGFRα-positive cells/mm^2^ were calculated for each section, and data from three sections were averaged. Data are expressed as mean ± standard error.

#### Transmission electron microscopy

Animals were sacrificed and perfused with saline followed by a fixation solution of 2.5% glutaraldehyde in 0.1 M phosphate buffer (PB; pH 7.4). Brains were sliced into 1 mm sections in coronal position with Rodent Brain matrix for adult mouse (30 g, coronal; 15003; TED PELLA, Inc.). The sections were further trimmed into small blocks (1x1x2 mm^3^) containing the central cross-sections of the corpus callosum. Sections including forebrain were post-fixed overnight at 4°C in the above fixative. After washing in 0.1 M PB, blocks were post-fixed with 1% osmium tetroxide (OsO4) in 0.1 M PB and then embedded with Epoxy resin. Embedded blocks were sectioned in midsagittal plane, oriented to visualize the cross-section of axons in corpus callosum. Ultrathin sections (50–60 nm) were cut with an ultramicrotome (LKB-I), stained with 3% uranyl acetate and lead citrate and examined with a transmission electron microscope (PHILIPS, CM-120).

For image analysis, six pictures from six fields were randomly taken at the site of corpus callosum for each mouse. Normal myelinated axons, demyelinated axons and remyelinated axons (characterized by thin myelin sheaths compared with normal myelinated fibers) were manually counted in a blinded manner by an independent person. Data are shown as remyelinated axons (average from six fields)/100 μm^2^ and total axon number (normal myelinated axons + demyelinated axon + remyelination axons) x (average from six fields)/100 μm^2^, which were determined from counting of 60–100 axons per picture in the central corpus callosum of forebrain of five mice from each treatment group.

### HRH3 genetic analysis

Protein coding, 5’ and 3’ untranslated regions and exon-intron boundaries of the *HRH3* gene were sequenced in 632 MS cases from the GeneMSA collection [21] and 2406 controls as part of a drug target sequencing study [22]. Two common (>5% minor allele frequency) exonic single nucleotide polymorphisms (SNPs), rs3787429 (p.332S) and rs3787430 (p.P326P), from this study were tested for association with MS susceptibility in GeneMSA cases and controls. Only rs3787429 showed nominal association (p<0.05) initially and so this result was followed up. Using reference haplotypes from the gene sequencing study, rs3787429 was imputed into two MS genome-wide association study (GWAS) datasets using the Beagle program [23]. Statistical analysis of allele- and genotype frequencies was performed using logistic regression. For GeneMSA, directly ascertained genotypes were available. For ANZgene [24] and IMSGC [25] collections, imputed data was used, with allelic dosage (additive model) used to infer allele frequencies and most likely genotype (recessive model), respectively. For both models, after imputation, the effective sample size was ~80% of the original. Nominal association with MS risk was observed for rs3787429 under both additive and recessive models, but genotype effect sizes were stronger under a recessive model in all three collections. These data are described in the results section.

### Statistical analysis

Unpaired t-Test or one-way analysis of variance was used to analyze differences between groups where appropriate. Graphs were generated using GraphPad5 PRISM software (GraphPad Software, Inco, San Diego, CA, USA).

## Results

### Phenotypic screening and investigation of OPC differentiation

**Fig 1:**
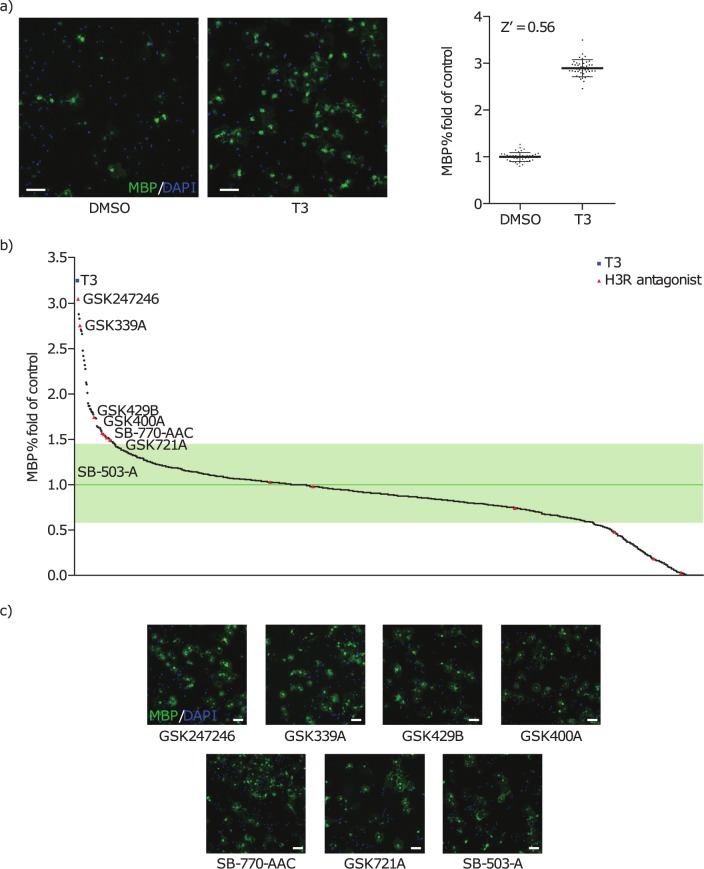
OPC differentiation assay. (a) Immunofluorescence images showing MBP (green) and DAPI (blue) staining in OPCs treated with DMSO (vehicle control 0.1%) or T3 (15 nM) (n = 1); (b) percentage fold change from control in MBP staining in OPCs treated with ~1000 compounds (15 nM) from GSK-proprietary annotated libraries, (n = 1); (c) Representative immunofluorescence images showing MBP (green) and DAPI (blue staining) in OPCs treated with selected H3R antagonists (1 μM) (n = 1) (scale bar = 100 μm). DAPI, 4',6-diamidino-2-phenylindole; DMSO, dimethyl sulfoxide; H3R, histamine receptor-3; MBP, myelin basic protein; OPC, OPC, oligodendrocyte precursor cell; T3, Triiodothyronine.

Agents that can promote OPC differentiation in preclinical models are thought to have the potential to restore halted remyelination in patients with MS [7]. In order to identify chemical leads and therapeutic targets, we established an *in vitro* phenotypic screening assay that measures the differentiation of rat primary OPCs. T3 was used as a positive control (Fig 1A, Z’ factor = 0.56), to demonstrate that the assay was robust. The assay was set up as a 384-well template with a capacity of up to 3000 compounds per round. Phenotypic screening of GSK-proprietary annotated libraries identified 36 compounds that promoted OPC differentiation in a dose-dependent manner. The results from one library comprising ~1000 candidates are illustrated in Fig 1B. Of the 36 compounds identified, seven were antagonists of H3R which demonstrated the ability to promote OPC differentiation (Fig 1C), suggesting involvement of H3R in regulating OPC differentiation.

**Fig 2:**
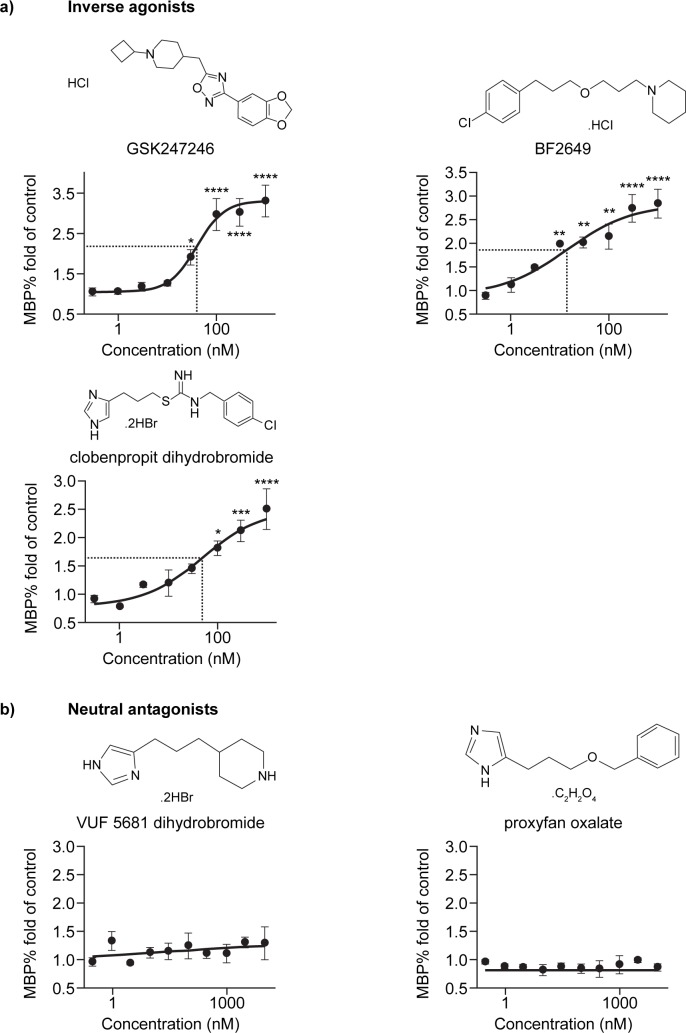
Effect of H3R antagonism on OPC differentiation. Percentage fold change from control in MBP staining in OPCs treated with H3R inverse agonists (a), GSK247246 (n = 9), BF2649 (n = 3), and clobenpropit (n = 3), or H3R neutral antagonists (b), VUF 5681 dihydrobromide (n = 4) and proxyfan oxalate (n = 3), over a range of nanomolar concentrations (0.3, 1, 3, 10, 30, 100, 300, 1000 nM). H3R, histamine receptor-3; MBP, myelin basic protein; p-values were generated by one-way ANOVA with post-hoc Dunnett's multiple comparisons test (GSK247246 (F = 16.77), BF2649 (F = 15.91), clobenpropit (F = 12.64), VUF 5681 dihydrobromide (F = 0.8353) and proxyfan oxalate (F = 0.5737), *p<0.05; **p<0.01; ***p<0.001; ****p<0.0001.

To validate H3R as a target for promotion of OPC differentiation, five well-characterized H3R tool compounds with diverse structures were selected for use in the OPC differentiation assay. Of these, three were inverse agonists, all of which promoted OPC differentiation in a dose-dependent manner with nanomolar potency (p<0.05; Fig 2A). In contrast, the remaining two compounds, which were neutral antagonists, showed no effect on OPC differentiation (Fig 2B). Both inverse agonists and neutral antagonists had no effect on OPC proliferation, as indicated by the unchanged total cell numbers across different treatment groups (S1 Fig). The differential effects on OPC differentiation between inverse agonists and neutral antagonists suggest that the constitutive activity of H3R negatively regulates the oligodendrocyte differentiation process.

### H3R expression in oligodendroglia and function in regulating oligodendrocyte differentiation

#### Cultured cells

**Fig 3:**
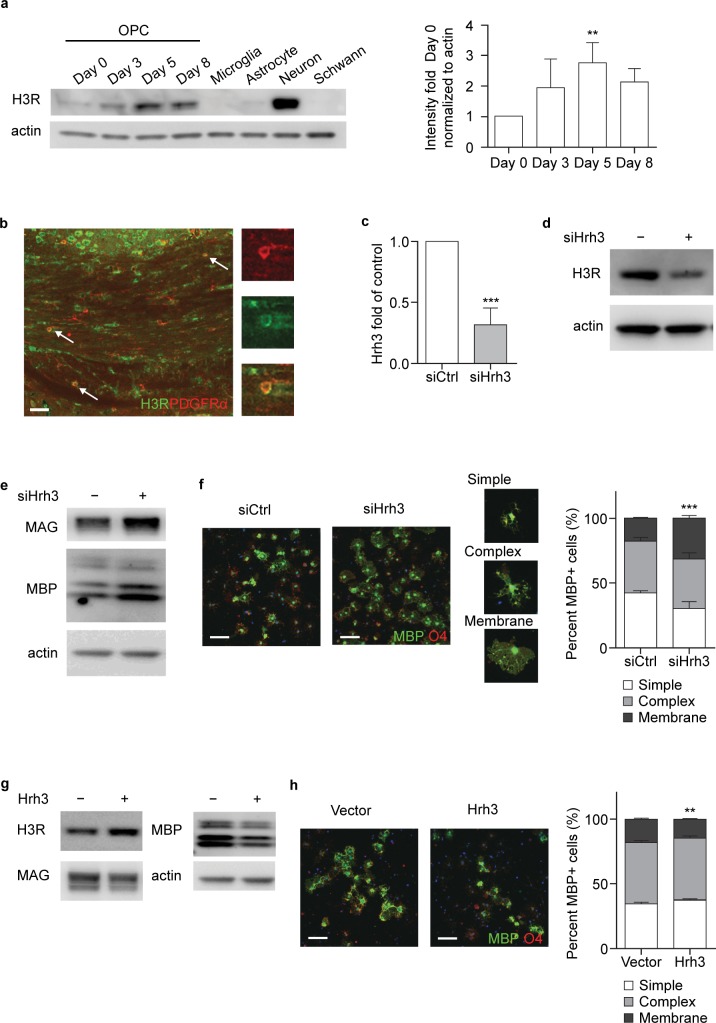
H3R negatively regulates oligodendrocyte differentiation. (a) Western blot analysis of H3R expression in OPCs on Days 0, 3, 5, and 8 of differentiation, and in microglia, astrocyte, neuron and Schwann cells (n = 3); Bar graph: Quantification of H3R expression level in OPCs on Days 0, 3, 5, and 8 of differentiation in Fig 3A. p-values were generated by one-way ANOVA with post-hoc Fisher's LSD (F = 4.045). **p<0.01. (b) representative immunofluorescence image showing H3R (green) and PDGFRα (red) staining in corpus callosum of naïve mouse brain (arrows indicate co-localization) (n = 2) (scale bar for large image = 50 μm); (c) RT-PCR of H3R gene expression in OPCs after gene knockdown (n = 8); p-values were generated by unpaired Student’s t-Test; (d) Western blot analysis of H3R expression in OPCs after gene knockdown (n = 3); (e) Western blot analysis of MAG and MBP expression in OPCs after gene knockdown (n = 4); (f) representative immunofluorescence images showing MBP (green) and O4 (red) staining in differentiating OPCs after gene knockdown (percentage of cells with simple, complex and membrane-like morphology was presented) (n = 4) (scale bar = 100 μm); The percentage of cells with membrane-like morphology among all O4-positive cells was compared and p-values were generated by unpaired Student’s t-Test; (g) Western blot analysis of MAG and MBP expression in OPCs after H3R overexpression (n = 3); (h) representative immunofluorescence images showing MBP (green) and O4 (red) staining in differentiating OPCs after Hrh3 overexpression (percentage of cells with simple, complex and membrane-like morphology is presented) (n = 3); The percentage of cells with membrane-like morphology among all O4-positive cells was compared and p-values were generated by unpaired Student’s t-Test (scale bar = 100 μm). H3R, histamine receptor-3; HRH3, histamine H3 receptor gene; MAG, myelin associated glycoprotein; MBP, myelin basic protein; O4, oligodendrocyte O-antigen 4; OPC, oligodendrocyte precursor cell; PDGFRα, platelet-derived growth factor receptor; RT-PCR, reverse transcriptase polymerase chain reaction; siRNA, small interfering RNA. **p<0.01; ***p<0.001.

Western blot analysis showed that H3R was highly expressed in neurons, minimally expressed in astrocytes/microglia/Schwann cells and moderately expressed in oligodendrocytes (Fig 3A). H3R could be detected at all stages of OPC differentiation, as shown by the representative western blot and quantitative analysis results, H3R was upregulated transiently during OPC lineage progression (on Day 3 and Day 5) and then downregulated on Day 8 (Fig 3A). Immunofluorescence staining demonstrated co-localization of H3R and PDGFRα, an OPC marker, in corpus callosum of naïve mouse brain, this result further confirmed H3R expression on OPCs. (Fig 3B).

To complement our pharmacological results and further address the function of H3R in OPC differentiation, endogenous H3R expression was knocked down via siRNA. siRNA knockdown of H3R in OPCs reduced endogenous expression down to 45% of control (Fig 3C and 3D). H3R knockdown led to an increase in the expression of the oligodendrocyte differentiation markers MAG and MBP (Fig 3E). Immunofluorescence staining with anti-MBP and anti-O4 antibodies revealed that the number of mature oligodendrocytes with membrane-like morphology was significantly increased (p<0.01) in cells in which H3R had been knocked down (Fig 3F). Moreover, overexpression of full-length H3R resulted in lower expression of the differentiation markers MBP and MAG, and fewer cells with mature morphology (Fig 3G and 3H), indicating fewer mature oligodendrocytes. These results indicated that increased expression of H3R alone, in the absence of the ligand histamine, is sufficient to suppress oligodendrocyte differentiation. Taken together, these findings suggest a negative role of constitutive H3R activity in oligodendrocyte differentiation.

#### MS lesions

**Fig 4:**
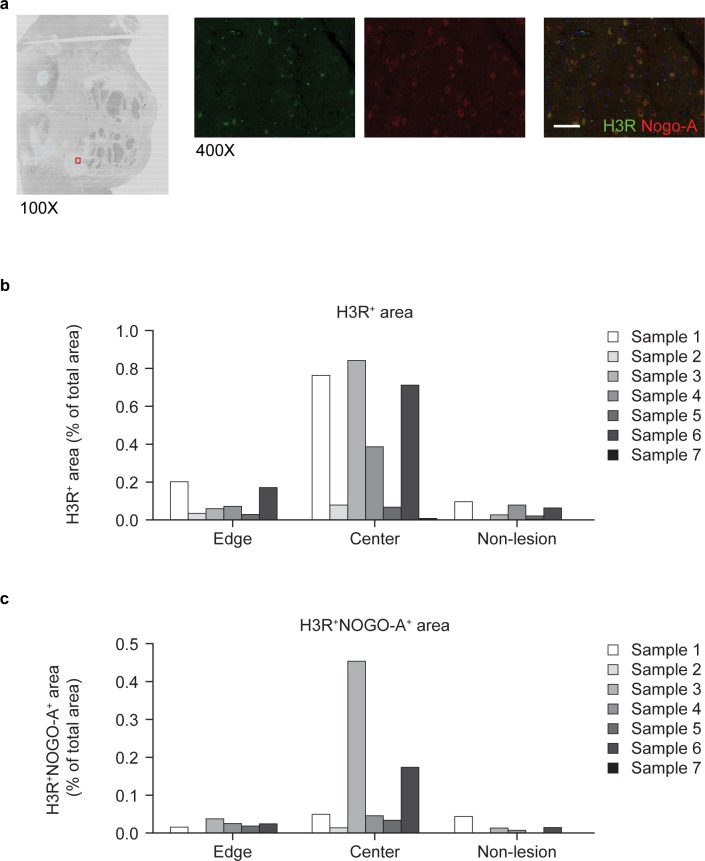
Expression of H3R in MS lesions from post-mortem brain sections. (a) Representative immunohistochemistry (using anti-MBP antibody) and immunofluorescence images showing H3R (green) and Nogo-A (red) staining in human post-mortem brain section (white matter in the brain stem) from patients with MS, scale bar = 50 μm; (b) and (c) quantitative analysis of edge, central and non-lesion localization of H3R (b) and H3R co-localized with Nogo-A (c). (n = 7). H3R, histamine receptor-3; MS, multiple sclerosis; Nogo-A, neurite outgrowth inhibitor-A.

Immunochemical staining of human post-mortem brain sections from patients with MS demonstrated expression of H3R in Nogo-A-positive oligodendrocytes in the center of different types of MS lesions (active/chronic active, shadow and grey matter lesions [GM]) (Fig 4A). Quantitative analysis confirmed that H3R staining alone and co-localized H3R/Nogo-A staining were higher in the central lesion compared with the lesion edge or non-lesion regions (Fig 4B and 4C). These results suggest increased H3R expression in demyelination areas.

### Molecular mechanism underlying the regulation of OPC differentiation by H3R

**Fig 5:**
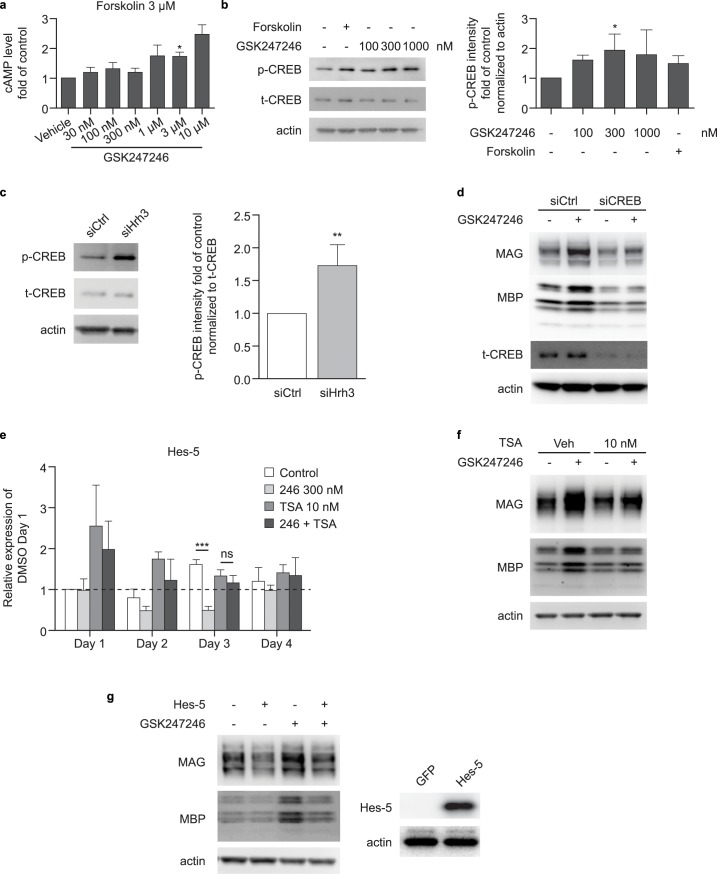
HR3 regulates oligodendrocyte differentiation through a cAMP/CREB/HDAC1/Hes-5 pathway. (a) Fold change in cAMP level in OPCs treated with 3 μM forskolin and 30 nM–10 μM GSK247246 (n = 4); p-values were generated by unpaired Student’s t-Test; (b) Western blot analysis of CREB expression in OPCs treated with 100 nM–1 μM GSK247246 (n = 3); p-values were generated by one-way ANOVA with post-hoc Fisher's LSD (F = 1.787); (c) Western blot analysis of CREB expression in OPCs after HRH3 gene knockdown (n = 5); p-values were generated by unpaired Student’s t-Test; (d) Western blot analysis of MAG and MBP expression in OPCs of CREB siRNA knockdown, treated with 300 nM GSK247246 or vehicle (n = 4); (e) quantitative RT-PCR showing the time course of Hes-5 expression in OPCs treated with 300 nM GSK247246, 10 nM TSA, or GSK247246 + TSA for 4 days (red line indicates baseline Hes-5 expression on Day 1) (n = 4); p-values are generated by two-way ANOVA with post-hoc Bonferroni's multiple comparisons test; (f) Western blot analysis of MAG and MBP expression in OPCs treated with 300 nM GSK247246, 10 nM TSA, or GSK247246 + TSA (n = 3); (g) Western blot analysis of MAG and MBP expression in OPCs in which Hes-5 is overexpressed, treated with 300 nM GSK247246 (n = 3). cAMP, cyclic adenosine monophosphate; CREB, cAMP response element-binding protein; H3R, histamine receptor-3; HRH3, histamine H3 receptor gene; HDAC1, histone deacetylase-1; MAG, myelin associated glycoprotein; MBP, myelin basic protein; OPC, oligodendrocyte precursor cell; PCR, polymerase chain reaction; p-CREB, phosphorylated CREB; siRNA, small interfering RNA; t-CREB, total CREB; TSA, trichostatin A. *p<0.05; **p<0.01; ***p<0.001.

Both the adenylyl cyclase/cAMP/CREB pathway and cPLA_2_ are involved in the downstream cascade of H3R constitutive activity [9]. Based on an initial observation that GSK247246 induced marked increases in intracellular cAMP and downstream CREB phosphorylation in a concentration-dependent manner in OPCs (p<0.05; Fig 5A and 5B), the underlying signaling mechanism involved in oligodendrocyte differentiation was investigated. Knockdown of H3R in these cells significantly increased CREB phosphorylation (p<0.01; Fig 5C), and knockdown of CREB attenuated the up-regulation of mature markers, MAG and MBP, induced by H3R inverse agonism (Fig 5D).

Gene profiling with OPCs treated with GSK247246 identified Hes-5 as a critical downstream gene in OPC differentiation, regulated by the activity of H3R. Quantitative PCR confirmed a reduction in Hes-5 expression in GSK247246-treated OPCs (p<0.001 on Day 3), which corresponds with the expression profile of H3R in differentiating OPCs. The reduction in Hes-5 expression by GSK247246 was attenuated by Trichostatin A (TSA), a selective inhibitor for histone deacetylase 1 (HDAC1; a negative regulator of Hes-5) (Fig 5E). Increased expression of MAG and MBP, induced by GSK247246, could also be diminished by co-treatment with TSA (Fig 5F). Finally, Hes-5 overexpression attenuated the expression of MAG and MBP in OPCs treated with GSK247246 (Fig 5G), which suggests a central role of Hes-5 in H3R regulation of OPC differentiation. Collectively, these results indicate that the effect of GSK247246 on OPC differentiation is mediated through the cAMP/CREB/HDAC1/Hes-5 pathway.

### *In vivo* efficacy of GSK247246 in mouse models of demyelination

**Fig 6:**
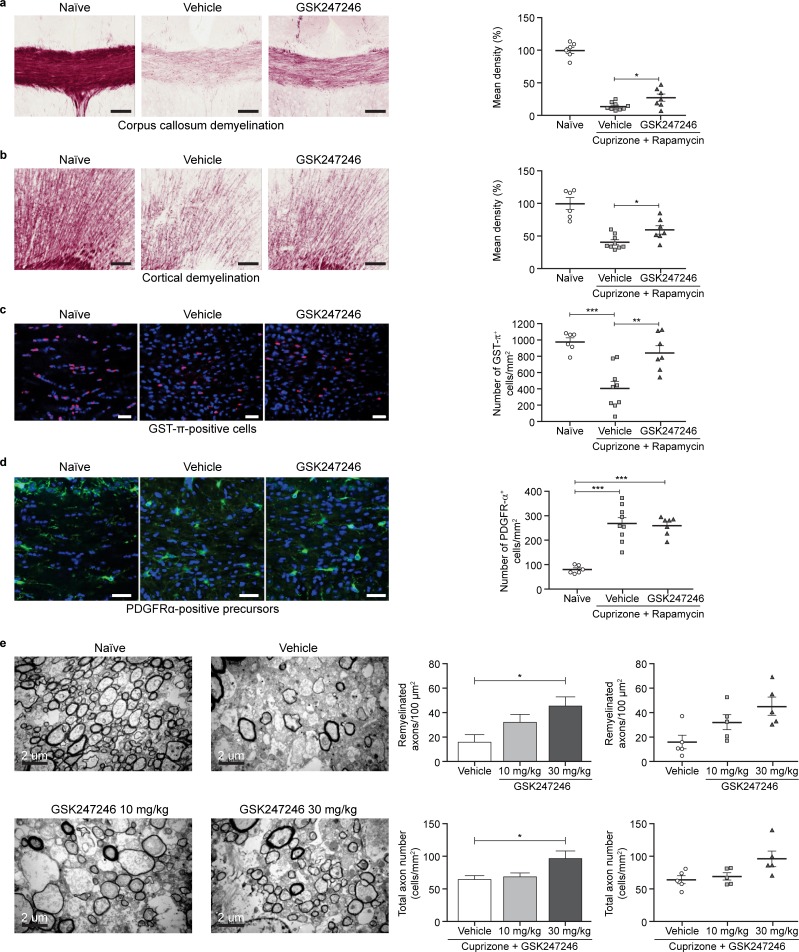
GSK247246 promotes remyelination in cuprizone mouse model. Histopathology of corpus callosum. Black gold staining of corpus callosum (a) and cortex (b) from cuprizone mice, untreated (n = 6) or treated with vehicle (n = 9) or 30 mg/kg GSK247246 (n = 7) for 9 days prior to sacrifice(scale bar = 100 μm); p-values were generated by unpaired Student’s t-Test; (c) representative immunofluorescence image showing GST-π (red) and DAPI (blue) staining in the corpus callosum, untreated (n = 6) or treated with vehicle (n = 9) or 30 mg/kg GSK247246 (n = 7) for 9 days prior to sacrifice (scale bar = 300 μm); p-values were generated by one-way ANOVA with post-hoc Bonferroni's multiple comparisons test (F = 14.59); (d) representative immunofluorescence image showing PDGFRα (green) staining in the corpus callosum, untreated (n = 6) or treated with vehicle (n = 9) or 30 mg/kg GSK247246 (n = 7) for 9 days prior to sacrifice (scale bar = 300 μm); p-values were generated by one-way ANOVA with post-hoc Bonferroni's multiple comparisons test (F = 27.32); (e) electron microscopy of myelin and oligodendrocytes surrounding axons in the corpus callosum of cuprizone-treated mice, untreated (n = 3) or treated with vehicle (n = 5), 10 mg/kg GSK247246 (n = 5) or 30 mg/kg GSK247246 (n = 5) for 9 days prior to sacrifice (scale bar = 2 μm); p-values were generated by one-way ANOVA with post-hoc Bonferroni's multiple comparisons test (Upper (F = 5.312), Lower (F = 4.438). DAPI, 4',6-diamidino-2-phenylindole; GST-π, glutathione S-transferase π; H3R, histamine receptor-3; PDGFRα, platelet-derived growth factor receptor. *p<0.05; **p<0.01; ***p<0.005.

The effect of GSK247246 was further examined in a cuprizone/rapamycin-induced demyelination model, in which a therapeutic protocol was employed. Histopathology analyses of corpus callosum and cortex revealed prominent demyelination in the cuprizone/rapamycin mouse model (Fig 6A and 6B). GSK247246-treated mice showed increased intensity of myelin staining in both corpus callosum and cortex at Day 9 of recovery, compared with control mice (p<0.05; Fig 6A and 6B).

Mechanistically, quantitative image analysis of random fields showed that GSK247246-treated mice displayed significantly increased numbers of mature GST-π-positive oligodendrocytes in corpus callosum, from ~400 cells/mm^2^ in the vehicle group to ~800 cells/mm^2^ in the treatment group (p<0.01; Fig 6C), with no concomitant change in PDGFRα-positive precursors (Fig 6D). This finding validated the hypothesis that H3R inverse agonist treatment promoted remyelination by modulating oligodendrocyte differentiation, but not proliferation or migration.

Subsequently, two doses (10 mg/kg and 30 mg/kg) of GSK247246 were tested in a pure cuprizone model without rapamycin. Treatment with GSK247246 (30 mg/kg) significantly increased the number of newly formed myelin sheaths (p<0.05), which were thinner than the myelin sheaths in the naïve mice (p<0.05; Fig 6E, top panels). Notably, the total number of axons was higher in the compound-treated group (30 mg/kg) compared with the vehicle group (p<0.05; Fig 6E, right bottom panel); this result suggests that treatment with GSK247246 subsequently preserves axon integrity in the context of demyelination.

Collectively, these data demonstrate a significant treatment effect of GSK247246 in the cuprizone model by enhancing remyelination.

### Genetic association between an *HRH3* SNP and MS susceptibility

**Fig 7:**
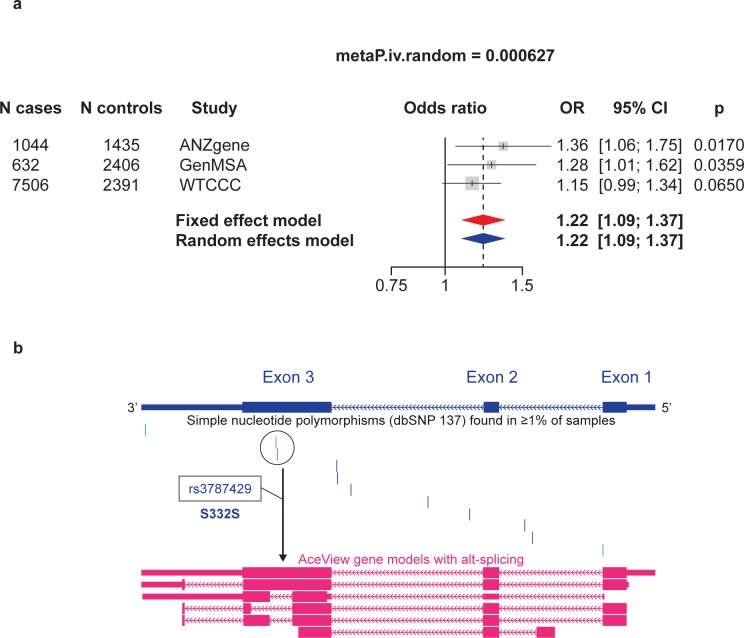
HRH3 SNP (p.S332S, rs3787429G>A) is associated with MS susceptibility. (a) Meta-analysis of rs3787429 G>A SNP association with MS risk in three collections: GeneMSA [21], ANZgene [24], and Wellcome Trust Case Control/IMSGC GWAS datasets [25]; (b)HRH3 gene structure showing the relative position of rs3787429 in exon 3 (vertical marks indicate position of other variants), and approximate location in an alternatively spliced segment of the HRH3 mRNA transcript. CI, confidence interval; HRH3, histamine H3 receptor gene; MS, multiple sclerosis; OR, odds ratio; SNP, single nucleotide polymorphism.

A common synonymous SNP in exon 3 (rs3787429 G>A, p.S332S) was nominally associated with MS susceptibility (odds ratio [OR] 1.28, 95% confidence interval (1.01–1.62), p = 0.0359) in the GeneMSA collection [21]). To validate this finding, rs3787429 was imputed into two GWAS datasets, from the ANZgene Consortium [24] and Wellcome Trust Case Control-International MS Genetics Consortium (WTCCC) [25], and tested for association using logistic regression. Meta-analysis across all three datasets, involving a combined sample size of 9182 cases and 6232 controls, revealed a consistent predisposing effect of AA genotype (OR 1.22 [1.09–1.37], meta p = 0.000627) (Fig 7A). In populations of North European ancestry (1000 genomes CEU population), rs3787429 G>A is not in linkage disequilibrium (r2>0.23) with any other SNP in the region, suggesting that it is the most plausible biological candidate SNP. rs3787429 is located in a variable region of the *HRH3* transcript that accounts for multiple known isoforms (Fig 7B).

## Discussion

As a member of the histamine receptor family highly expressed in the CNS, H3R has been identified as a drug target for the potential treatment of neurological and psychiatric disorders and its function in neurons has been well characterized. This G_i/o_ protein-coupled receptor is hypothesized to moderate the synthesis and release of histamine and other neurotransmitters from neuron synapses by inhibiting the cAMP-dependent protein kinase cascade and the calcium/calmodulin pathway [10]; however, its role in other neural cell types remains unclear. Here, findings from a high-throughput quantitative primary OPC screening assay, which were further supported by results of characterization of H3R expression and function in oligodendroglial lineage and gene manipulation, suggest that H3R may be a potential therapeutic target for promotion of remyelination in MS.

### The role of H3R in oligodendrocyte differentiation and remyelination

This study is the first to demonstrate a critical role for H3R in OPC differentiation. *In vitro* and *in vivo* evidence consistently demonstrated an inhibitory role for H3R in OPC differentiation, with its constitutive activity central to the rate of remyelination.

A phenotypic screening assay was established initially with the aim of identifying novel drug targets to promote remyelination. Phenotypic screening has been proven to be a robust method of identifying novel drug targets [26]. Indeed, a recent paper reported that a compound identified through such a phenotypic screening strategy, Benztropine, subsequently demonstrated efficacy in a series of MS-related animal models [27]. Here, we utilized a collection of various highly selective compounds in our OPC screen, which offered us the opportunity to reposition compounds or tractable drug targets that have not been previously studied extensively into the therapeutic area of myelination. With seven positive hits closely related to one target, the results of the phenotypic screening identified H3R as a potential target for remyelination.

H3R is one of the few examples for which constitutive G-protein-coupled receptor signaling has been shown to occur both *in vitro* and *in vivo* [28]. Given that OPCs and oligodendrocytes do not express histidine decarboxylase, an enzyme critical for histamine biosynthesis, the OPC culture system does not contain the natural H3R ligand histamine. However, four inverse agonists of H3R with diverse structures were able to promote OPC differentiation within the culture system while two neutral antagonists showed no effect. This suggests that the presence of H3R alone negatively regulates OPC differentiation, supporting constitutive activity in the absence of histamine. Moreover, H3R knockdown in this culture system promoted differentiation, as demonstrated by increased expression of differentiation markers MAG and MBP, and increased the number of mature oligodendrocytes with membrane-like morphology, while H3R overexpression had the converse effect. Importantly, H3R expression is transiently upregulated in pre-myelinating oligodendrocytes and subsequently downregulated to allow cell terminal maturation. This is consistent with a role in the decreased rate of myelin repair seen in MS lesions, whereby abundant OPCs, which express H3R on their surface, may be blocked in the pre-myelinating stage. In the post-mortem brain sections of patients with MS, H3R expression alone and co-localized with Nogo-A was found to be higher in the center of white matter demyelination lesions compared with the lesion edge or non-lesion areas, suggesting H3R is expressed in demyelination lesions.

Importantly, a role for H3R in remyelination was supported by *in vivo* demyelination models, in which H3R antagonism with GSK247246 increased myelin expression and mature GST-π-positive oligodendrocytes. Moreover, in a second demyelination model, the number of newly formed myelin sheaths and the total number of axons were both increased by GSK247246 treatment.

### The role of H3R in the cAMP/CREB pathway

It has previously been demonstrated that oligodendrocyte differentiation is regulated by cAMP and CREB [29, 30]; however, the upstream and downstream signaling surrounding these proteins remains unclear. We further characterized H3R activity by investigating the signaling mechanism involved in negative regulation of OPC differentiation, focusing on cAMP and CREB activity. This study provided evidence of correlation between H3R and cAMP/CREB activity in differentiating oligodendrocytes, whereby H3R inverse agonism with GSK247246 increased intracellular cAMP levels and phosphorylation of CREB; H3R knockdown consistently induced CREB phosphorylation. These results suggest that H3R has an inhibitory effect on the signaling pathway leading to CREB phosphorylation during oligodendrocyte differentiation. Importantly, knockdown of CREB in this system cancelled the effect of H3R antagonism, as demonstrated by inhibition of GSK247246-induced MAG and MBP expression increases. These results are in line with a study conducted by Morisset *et al*. that demonstrated constitutive activity of H3R in rodent brain, where histaminergic neuron activity was controlled via cAMP signaling [28].

**Fig 8:**
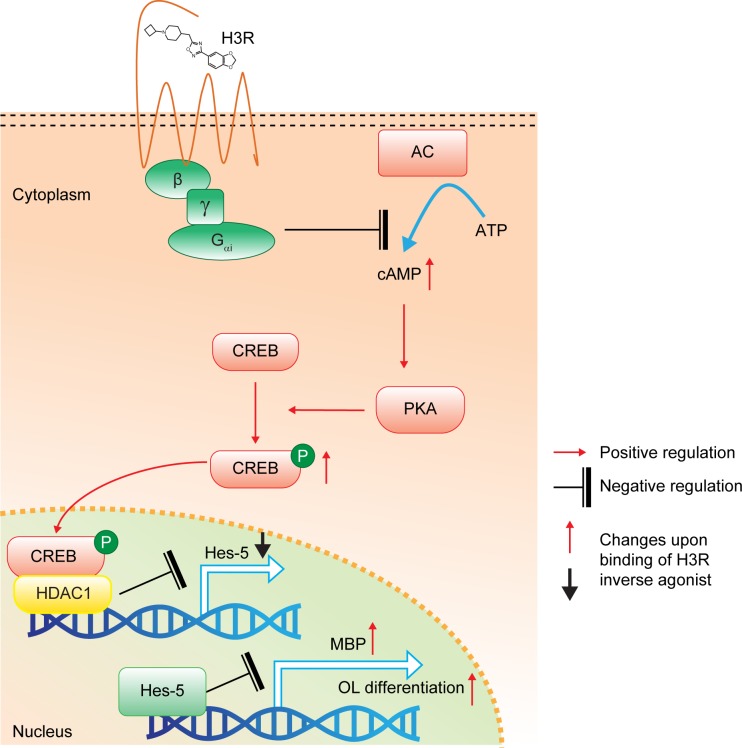
Schematic of downstream pathway of H3R in OPCs. Oligodendrocyte differentiation is regulated through the cAMP/CREB/HDAC1/Hes-5 pathway. Constitutively active H3R inhibits cAMP increase, which would otherwise activate PKA phosphorylation of CREB leading to oligodendrocyte differentiation through HDAC1 and Hes-5. AC, adenyl cyclase; ATP, adenosine triphosphate; cAMP, cyclic adenosine monophosphate, CREB, cAMP response elements; H3R, histamine receptor 3; HDAC, Histone deacetylase. MBP, myelin basic protein; OL, oligodendrocyte; OPC, oligodendrocyte precursor cell; P, phosphate; PKA, protein kinase A.

Hes-5 has been well characterized as a negative regulator of OPC differentiation and the transcription of Hes-5 is known to be negatively controlled by HDAC1. In this study, GSK247246 reduced Hes-5 expression in oligodendrocyte culture, an effect that was inhibited in the presence of the selective HDAC1 inhibitor TSA. Moreover, GSK247246-induced OPC differentiation was also inhibited in the presence of TSA, or by overexpression of Hes-5. Collectively, these results indicate that the effect of the H3R inverse agonist GSK247346 on OPC differentiation is mediated through the cAMP/CREB/HDAC1/Hes-5 pathway (Fig 8).

### H3R and *HRH3* have clinical relevance to patients with MS

Presently, the majority of MS therapies are immunomodulatory and immunosuppressive agents, which do not reverse disease progression. Mechanistically, the findings presented in this study support the hypothesis that H3R inverse agonist treatment modulates OPC differentiation and remyelination. Indeed, high expression of H3R was observed in oligodendroglial cells in demyelinated lesions in brain samples of patients with MS; Furthermore, a common *HRH3* SNP was found to be suggestively associated with susceptibility to MS in the present study. A possible explanation for why the *HRH3*-MS association has not been previously observed in large MS GWAS is that in the collections analyzed, rs3787429 appears to exert a genotype (recessive) effect rather than an allelic effect, and most GWAS analyses are undertaken assuming the latter.

In a recent Phase II, randomized, placebo-controlled study of 131 patients with relapsing-remitting MS over 48 weeks of treatment, treatment with the H3R inverse agonist GSK239512 was shown to produce a small but positive effect on remyelination. This suggests that the efficacy of H3R inverse agonists in preclinical models of MS translates into patients with MS [31]. As such, restoration of myelin repair over the long term could be a promising regenerative strategy to halt disability/disease progression in patients with MS [32]. Given the complexity of MS, it is noteworthy that H3R has been reported to play an important role in maintaining blood brain barrier integrity during neuroinflammation in a model of experimental autoimmune encephalomyelitis[33]; Consequently, H3R agonism has been proposed as a potential protective factor against MS progression.[34] Therefore in a clinical setting, the therapeutic window for H3R inverse agonist administration and target patient population should be carefully selected to avoid any potential side effect on blood brain barrier integrity.

In conclusion, H3R was identified as a novel target for promotion of oligodendrocyte differentiation and remyelination in patients with MS via phenotypic screening, then validated by results of *in vitro and in vivo* models, and pathology of human samples of patients with MS. These results provide support for a role of H3R in the promotion of CNS myelin repair through increased OPC differentiation and maturation and therefore, as a potential route for addressing the unmet medical need for pro-remyelination treatments in MS and other demyelinating disease.

## Supporting information

S1 Fig Effect of H3R antagonism on OPC proliferation. Percentage fold change from control in total cell number in OPCs treated with H3R inverse agonists (a), GSK247246 (n = 6), BF2649 (n = 3), and clobenpropit (n = 3), or H3R neutral antagonists (b), VUF 5681 dihydrobromide (n = 4) and proxyfan oxalate (n = 3), over a range of nanomolar concentrations (0.3, 1, 3, 10, 30, 100, 300, 1000 nM). p-values were generated by one-way ANOVA with post-hoc Dunnett's multiple comparisons test (GSK247246 (F = 2.286), BF2649 (F = 12.84), clobenpropit (F = 1.033), VUF 5681 dihydrobromide (F = 0.7808) and proxyfan oxalate (F = 0.9899); ****p<0.0001.
(PDF)

S2 Fig Original blot images for all western blot experiments. (PDF)

S1 Table Original data used for the analysis of genetic association between an HRH3 SNP and MS susceptibility. (XLSX)

## References

[1] Browne P, Chandraratna D, Angood C, Tremlett H, Baker C, Taylor BV, et al Atlas of Multiple Sclerosis 2013: A growing global problem with widespread inequity. Neurology. 2014;83: 1022–1024. doi: 10.1212/WNL.0000000000000768 25200713 10.1212/WNL.0000000000000768 PMC4162299

[2] Kieseier BC, Hartung HP. Multiple paradigm shifts in multiple sclerosis. Curr Opin Neurol. 2003;16: 247–252. doi: 10.1097/01.wco.0000073923.19076.75 12858058 10.1097/01.wco.0000073923.19076.75

[3] Chang A, Tourtellotte WW, Rudick R, Trapp BD. Premyelinating oligodendrocytes in chronic lesions of multiple sclerosis. N Engl J Med. 2002;346: 165–173. doi: 10.1056/NEJMoa010994 11796850 10.1056/NEJMoa010994

[4] Kuhlmann T, Miron V, Cui Q, Wegner C, Antel J, Bruck W. Differentiation block of oligodendroglial progenitor cells as a cause for remyelination failure in chronic multiple sclerosis. Brain. 2008;131: 1749–1758. doi: 10.1093/brain/awn096 18515322 10.1093/brain/awn096

[5] Wolswijk G. Chronic stage multiple sclerosis lesions contain a relatively quiescent population of oligodendrocyte precursor cells. J Neurosci. 1998;18: 601–609. 9425002 10.1523/JNEUROSCI.18-02-00601.1998 PMC6792542

[6] Franklin RJ, ffrench-Constant C, Edgar JM, Smith KJ. Neuroprotection and repair in multiple sclerosis. Nat Rev Neurol. 2012;8: 624–634. doi: 10.1038/nrneurol.2012.200 23026979 10.1038/nrneurol.2012.200

[7] Franklin RJ, Ffrench-Constant C. Remyelination in the CNS: from biology to therapy. Nat Rev Neurosci. 2008;9: 839–855. doi: 10.1038/nrn2480 18931697 10.1038/nrn2480

[8] Tanaka T, Yoshida S. Mechanisms of remyelination: recent insight from experimental models. Biomol Concepts. 2014;5: 289–298. doi: 10.1515/bmc-2014-0015 25372760 10.1515/bmc-2014-0015

[9] Leurs R, Bakker RA, Timmerman H, de Esch IJ. The histamine H3 receptor: from gene cloning to H3 receptor drugs. Nat Rev Drug Discov. 2005;4: 107–120. doi: 10.1038/nrd1631 15665857 10.1038/nrd1631

[10] Passani MB, Blandina P. Histamine receptors in the CNS as targets for therapeutic intervention. Trends Pharmacol Sci. 2011;32: 242–249. doi: 10.1016/j.tips.2011.01.003 21324537 10.1016/j.tips.2011.01.003

[11] Grove RA, Harrington CM, Mahler A, Beresford I, Maruff P, Lowy MT, et al A randomized, double-blind, placebo-controlled, 16-week study of the H3 receptor antagonist, GSK239512 as a monotherapy in subjects with mild-to-moderate Alzheimer's disease. Curr Alzheimer Res. 2014;11: 47–58. 24359500 10.2174/1567205010666131212110148

[12] Jarskog LF, Lowy MT, Grove RA, Keefe RS, Horrigan JP, Ball MP, et al A Phase II study of a histamine H(3) receptor antagonist GSK239512 for cognitive impairment in stable schizophrenia subjects on antipsychotic therapy. Schizophr Res. 2015;164: 136–142. doi: 10.1016/j.schres.2015.01.041 25728831 10.1016/j.schres.2015.01.041

[13] Nathan PJ, Boardley R, Scott N, Berges A, Maruff P, Sivananthan T, et al The safety, tolerability, pharmacokinetics and cognitive effects of GSK239512, a selective histamine H(3) receptor antagonist in patients with mild to moderate Alzheimer's disease: a preliminary investigation. Curr Alzheimer Res. 2013;10: 240–251. 23521503 10.2174/1567205011310030003

[14] Chen Y, Balasubramaniyan V, Peng J, Hurlock EC, Tallquist M, Li J, et al Isolation and culture of rat and mouse oligodendrocyte precursor cells. Nat Protoc. 2007;2: 1044–1051. doi: 10.1038/nprot.2007.149 17546009 10.1038/nprot.2007.149

[15] Skaper SD, Argentini C, Barbierato M. Culture of neonatal rodent microglia, astrocytes, and oligodendrocytes from cortex and spinal cord. Methods Mol Biol. 2012;846: 67–77. doi: 10.1007/978-1-61779-536-7_7 22367802 10.1007/978-1-61779-536-7_7

[16] Tao Y. Isolation and culture of Schwann cells. Methods Mol Biol. 2013;1018: 93–104. doi: 10.1007/978-1-62703-444-9_9 23681620 10.1007/978-1-62703-444-9_9

[17] Dugas JC, Tai YC, Speed TP, Ngai J, Barres BA. Functional genomic analysis of oligodendrocyte differentiation. J Neurosci. 2006;26: 10967–10983. doi: 10.1523/JNEUROSCI.2572-06.2006 17065439 10.1523/JNEUROSCI.2572-06.2006 PMC6674672

[18] Armstrong RC, Le TQ, Frost EE, Borke RC, Vana AC. Absence of fibroblast growth factor 2 promotes oligodendroglial repopulation of demyelinated white matter. J Neurosci. 2002;22: 8574–8585. 12351731 10.1523/JNEUROSCI.22-19-08574.2002 PMC6757804

[19] Dutta R, Chomyk AM, Chang A, Ribaudo MV, Deckard SA, Doud MK, et al Hippocampal demyelination and memory dysfunction are associated with increased levels of the neuronal microRNA miR-124 and reduced AMPA receptors. Ann Neurol. 2013;73: 637–645. doi: 10.1002/ana.23860 23595422 10.1002/ana.23860 PMC3679350

[20] Franklin K, Paxinos G. The Mouse Brain in Stereotaxic Coordinates, 3rd Edition: Academic Press; 2007.

[21] Baranzini SE, Wang J, Gibson RA, Galwey N, Naegelin Y, Barkhof F, et al Genome-wide association analysis of susceptibility and clinical phenotype in multiple sclerosis. Hum Mol Genet. 2009;18: 767–778. doi: 10.1093/hmg/ddn388 19010793 10.1093/hmg/ddn388 PMC4334814

[22] Nelson MR, Wegmann D, Ehm MG, Kessner D, St Jean P, Verzilli C, et al An abundance of rare functional variants in 202 drug target genes sequenced in 14,002 people. Science. 2012;337: 100–104. doi: 10.1126/science.1217876 22604722 10.1126/science.1217876 PMC4319976

[23] Browning BL, Browning SR. A unified approach to genotype imputation and haplotype-phase inference for large data sets of trios and unrelated individuals. Am J Hum Genet. 2009;84: 210–223. doi: 10.1016/j.ajhg.2009.01.005 19200528 10.1016/j.ajhg.2009.01.005 PMC2668004

[24] Australia, New Zealand Multiple Sclerosis Genetics C. Genome-wide association study identifies new multiple sclerosis susceptibility loci on chromosomes 12 and 20. Nat Genet. 2009;41: 824–828. doi: 10.1038/ng.396 19525955 10.1038/ng.396

[25] International Multiple Sclerosis Genetics C, Wellcome Trust Case Control C, Sawcer S, Hellenthal G, Pirinen M, Spencer CC, et al Genetic risk and a primary role for cell-mediated immune mechanisms in multiple sclerosis. Nature. 2011;476: 214–219. doi: 10.1038/nature10251 21833088 10.1038/nature10251 PMC3182531

[26] Swinney DC, Anthony J. How were new medicines discovered? Nat Rev Drug Discov. 2011;10: 507–519. doi: 10.1038/nrd3480 21701501 10.1038/nrd3480

[27] Deshmukh VA, Tardif V, Lyssiotis CA, Green CC, Kerman B, Kim HJ, et al A regenerative approach to the treatment of multiple sclerosis. Nature. 2013;502: 327–332. doi: 10.1038/nature12647 24107995 10.1038/nature12647 PMC4431622

[28] Morisset S, Rouleau A, Ligneau X, Gbahou F, Tardivel-Lacombe J, Stark H, et al High constitutive activity of native H3 receptors regulates histamine neurons in brain. Nature. 2000;408: 860–864. doi: 10.1038/35048583 11130725 10.1038/35048583

[29] Raible DW, McMorris FA. Oligodendrocyte differentiation and progenitor cell proliferation are independently regulated by cyclic AMP. J Neurosci Res. 1993;34: 287–294. doi: 10.1002/jnr.490340305 8384267 10.1002/jnr.490340305

[30] Sato-Bigbee C, DeVries GH. Treatment of oligodendrocytes with antisense deoxyoligonucleotide directed against CREB mRNA: effect on the cyclic AMP-dependent induction of myelin basic protein expression. J Neurosci Res. 1996;46: 98–107. doi: 10.1002/(SICI)1097-4547(19961001)46:1&lt;98::AID-JNR12&gt;3.0.CO;2-8 8892110 10.1002/(SICI)1097-4547(19961001)46:1<98::AID-JNR12>3.0.CO;2-8

[31] Schwartzbach CJ, Grove RA, Brown R, Tompson D, Then Bergh F, Arnold DL. Lesion remyelinating activity of GSK239512 versus placebo in patients with relapsing-remitting multiple sclerosis: a randomised, single-blind, phase II study. J Neurol. 2016. 10.1007/s00415-016-8341-7 PMC5306088 27888416

[32] Chamberlain KA, Nanescu SE, Psachoulia K, Huang JK. Oligodendrocyte regeneration: Its significance in myelin replacement and neuroprotection in multiple sclerosis. Neuropharmacology. 2016;110: 633–643. doi: 10.1016/j.neuropharm.2015.10.010 26474658 10.1016/j.neuropharm.2015.10.010 PMC4841742

[33] Teuscher C, Subramanian M, Noubade R, Gao JF, Offner H, Zachary JF, et al Central histamine H3 receptor signaling negatively regulates susceptibility to autoimmune inflammatory disease of the CNS. Proc Natl Acad Sci U S A. 2007;104: 10146–10151. doi: 10.1073/pnas.0702291104 17548817 10.1073/pnas.0702291104 PMC1891222

[34] Jadidi-Niaragh F, Mirshafiey A. Histamine and histamine receptors in pathogenesis and treatment of multiple sclerosis. Neuropharmacology. 2010;59: 180–189. doi: 10.1016/j.neuropharm.2010.05.005 20493888 10.1016/j.neuropharm.2010.05.005

