# Supplementary figures and images for: Histamine Receptor 3 negatively regulates oligodendrocyte differentiation and remyelination
Source: PLoS One. 2017 Dec 18;12(12):e0189380. doi: 10.1371/journal.pone.0189380 (PMC5734789; doi:10.1371/journal.pone.0189380)

Figure S1

**a) Inverse agonists**

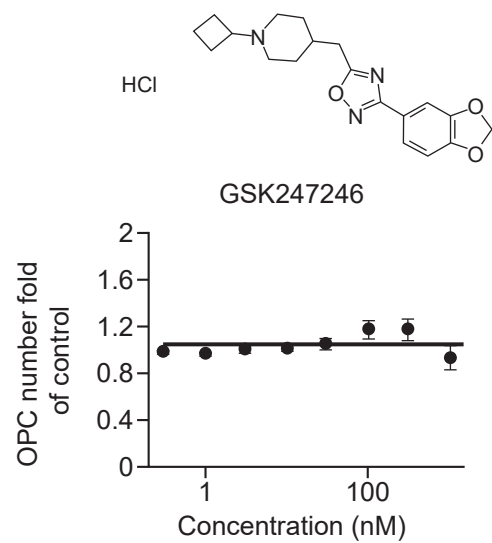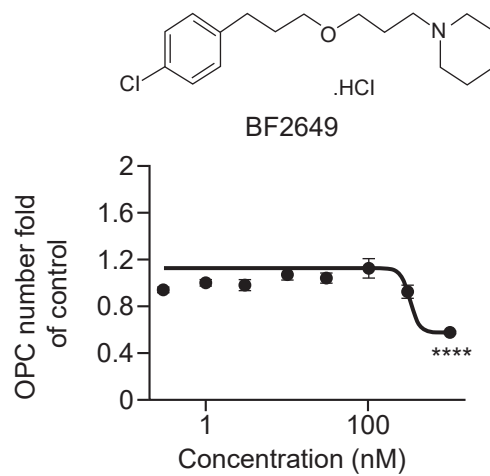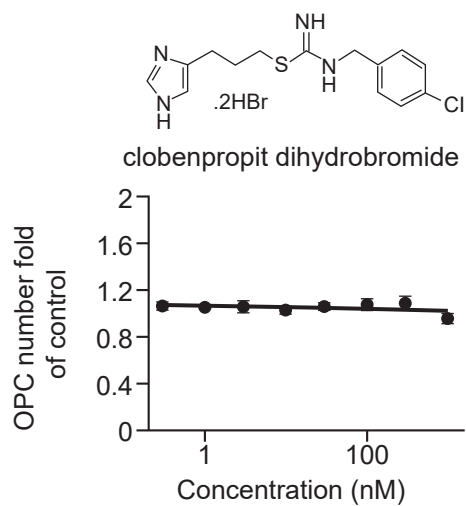

**b) Neutral antagonists**

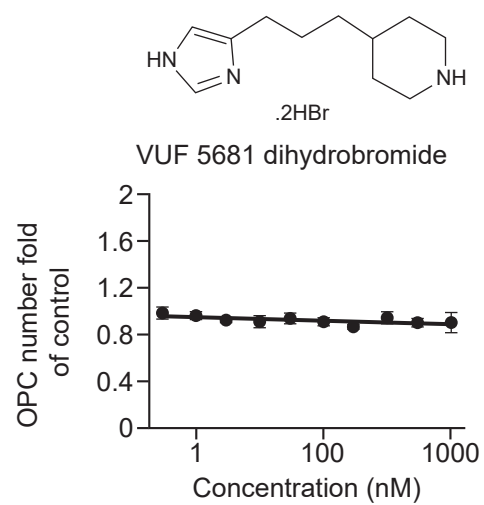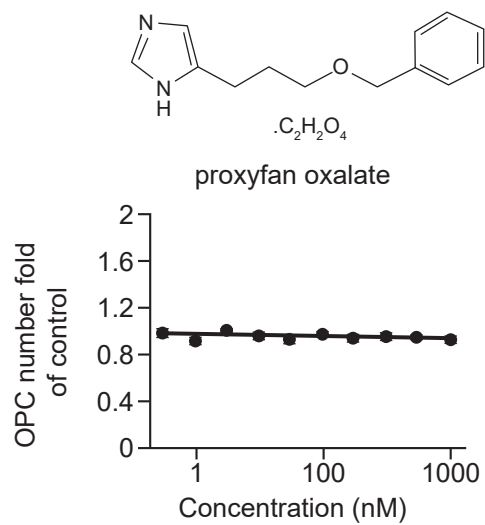

Supplement: S1 Fig — Percentage fold change from control in total cell number in OPCs treated with H3R inverse agonists (a), GSK247246 (n = 6), BF2649 (n = 3), and clobenpropit (n = 3), or H3R neutral antagonists (b), VUF 5681 dihydrobromide (n = 4) and proxyfan oxalate (n = 3), over a range of nanomolar concentrations (0.3, 1, 3, 10, 30, 100, 300, 1000 nM). p-values were generated by one-way ANOVA with post-hoc Dunnett's multiple comparisons test (GSK247246 (F = 2.286), BF2649 (F = 12.84), clobenpropit (F = 1.033), VUF 5681 dihydrobromide (F = 0.7808) and proxyfan oxalate (F = 0.9899); ****p<0.0001. (PDF) [file pone.0189380.s001.pdf]
