# Supplementary material for: Histamine Receptor 3 negatively regulates oligodendrocyte differentiation and remyelination
Source: PLoS One. 2017 Dec 18;12(12):e0189380. doi: 10.1371/journal.pone.0189380 (PMC5734789; doi:10.1371/journal.pone.0189380)

Figure 3

Fig. 3a

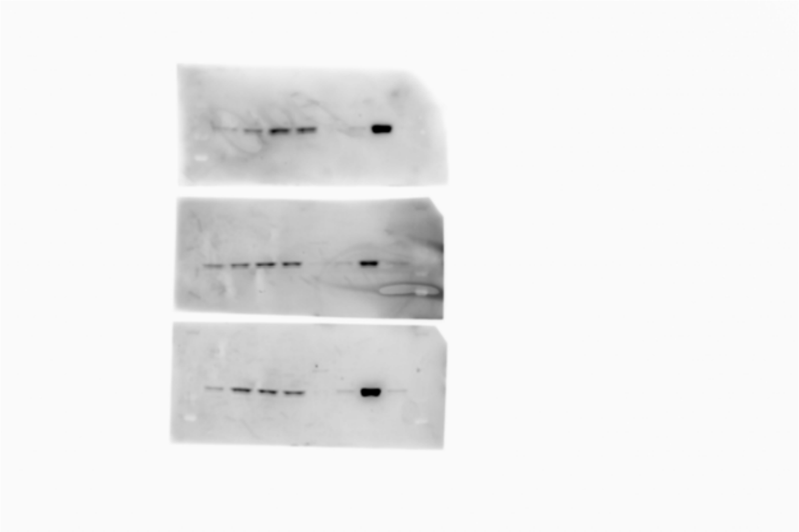

H3R

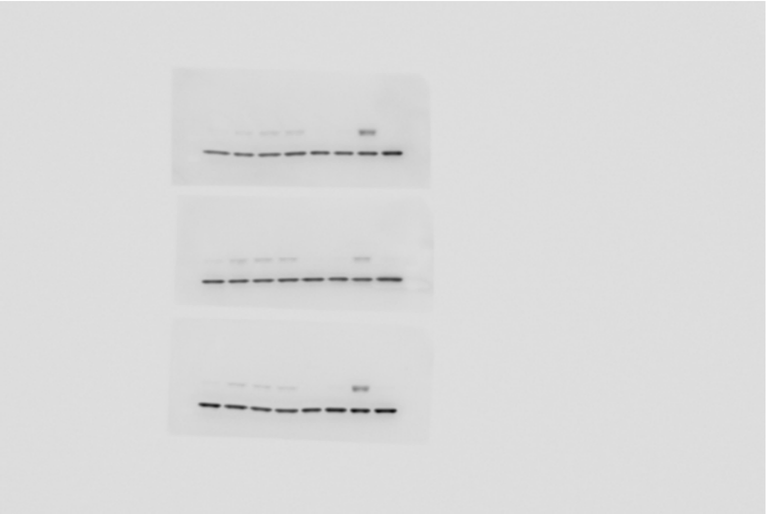

Actin

Fig. 3d

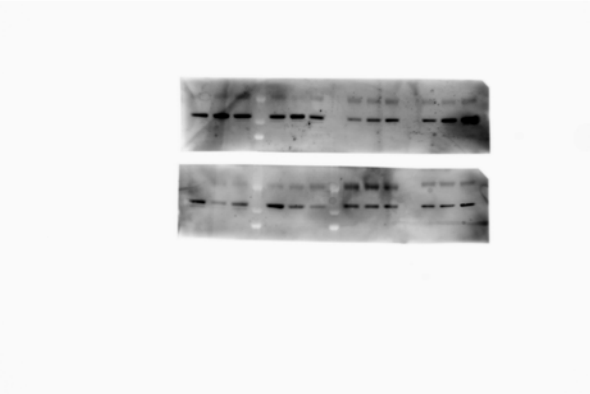

H3R

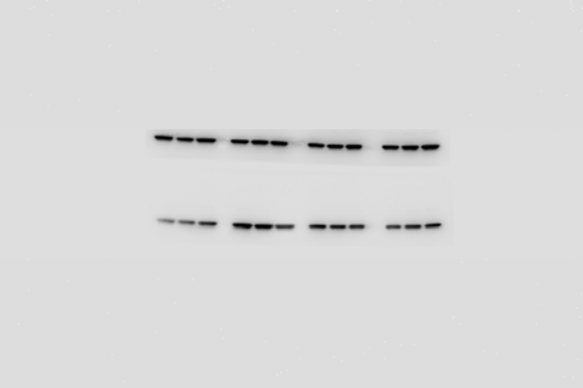

Actin

Fig. 3e

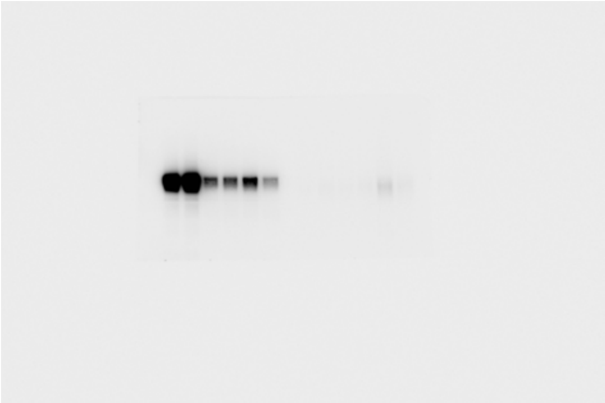

MAG

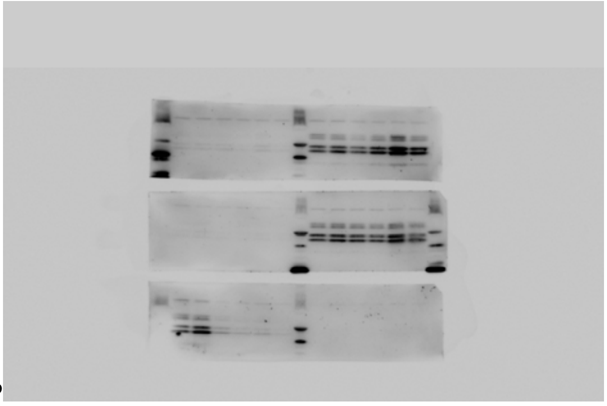

MBP

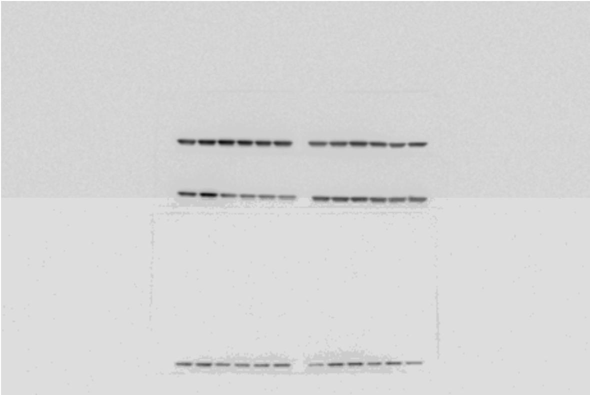

Actin

Fig. 3g

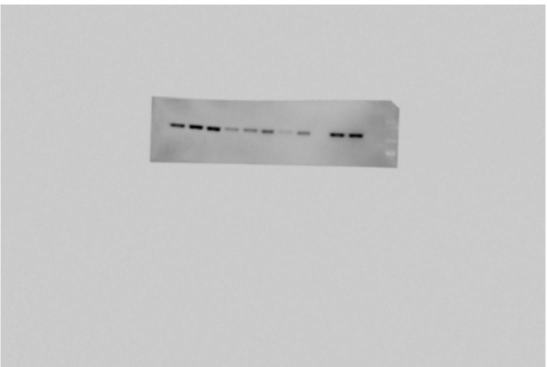

H3R

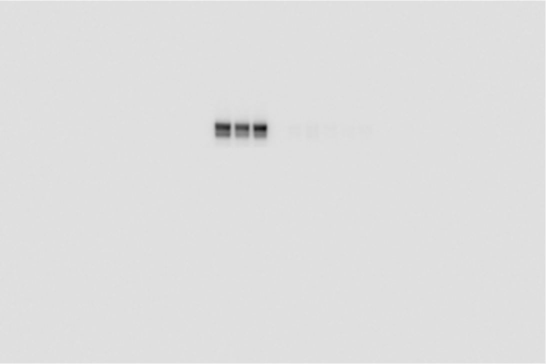

MAG

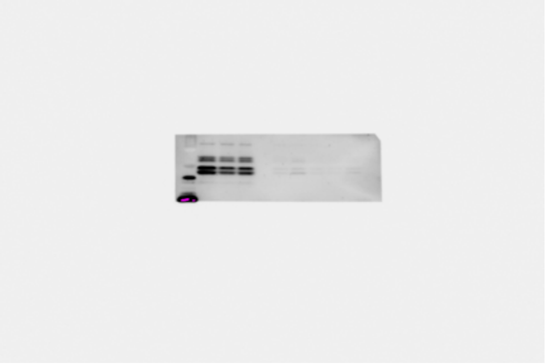

MBP

Actin

Figure 5

Fig. 5b

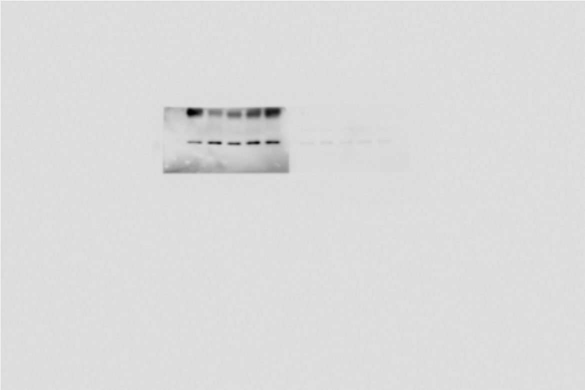

p-CREB

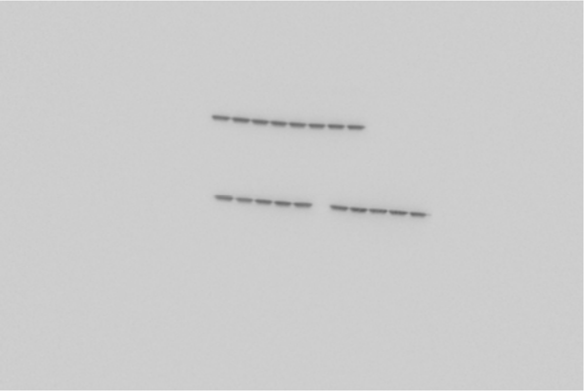

Actin

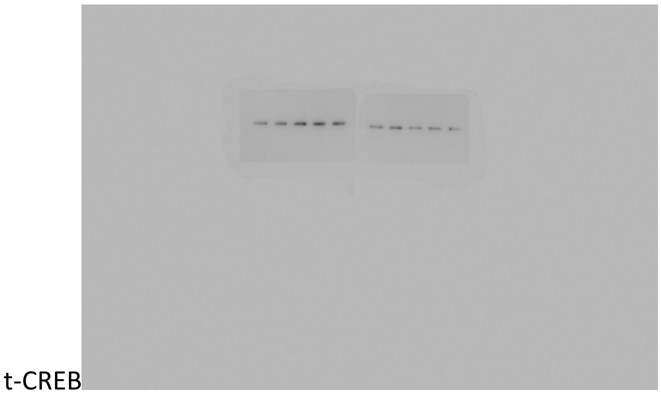

t-CREB

Fig. 5c

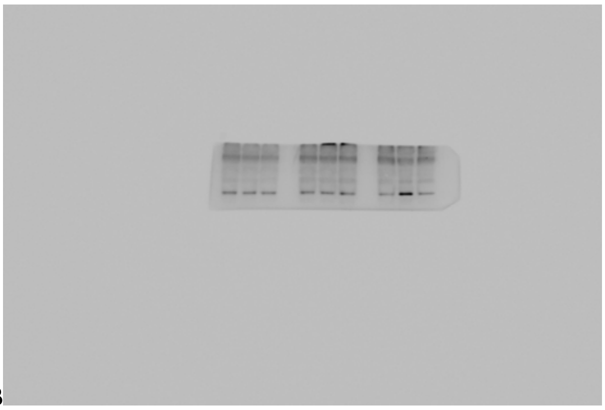

p-CREB

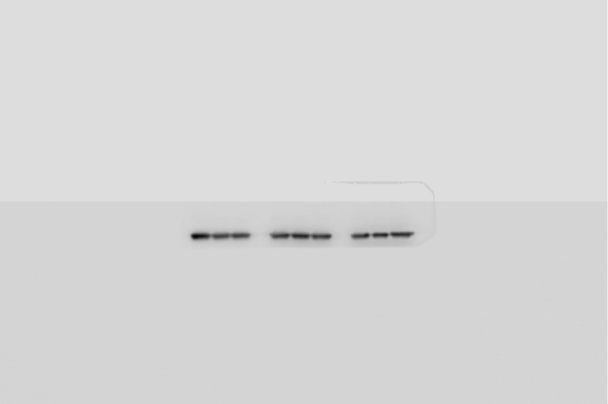

Actin

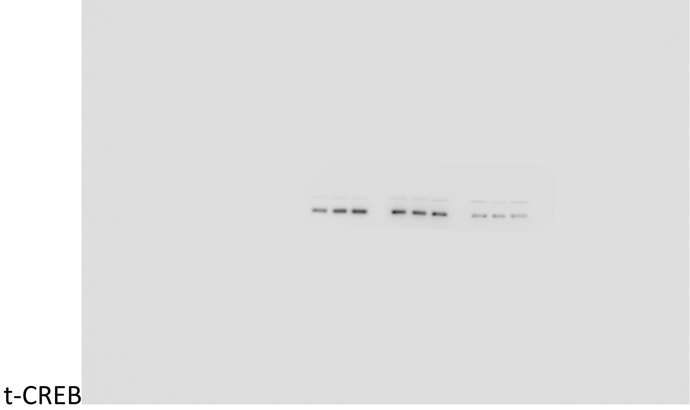

t-CREB

Fig. 5d

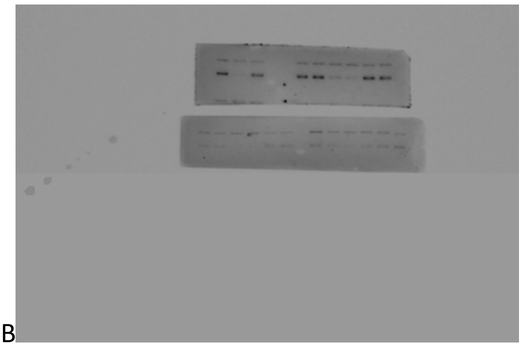

t-CREB

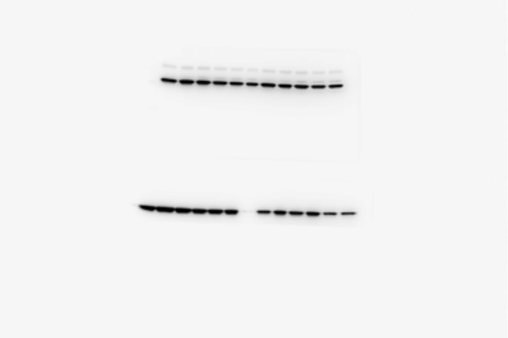

Actin

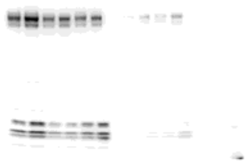

MAG

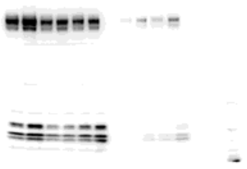

MBP

Figure 5f

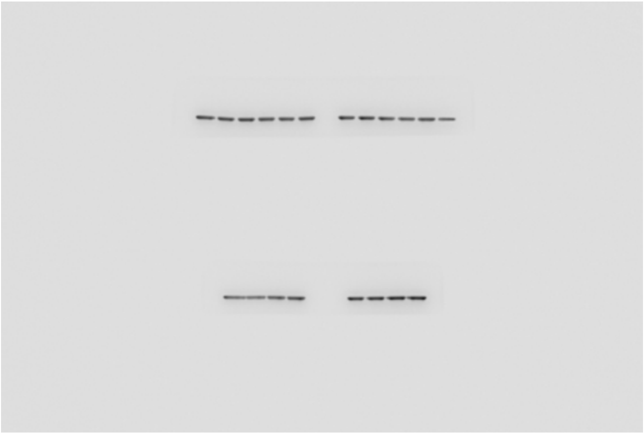

Actin

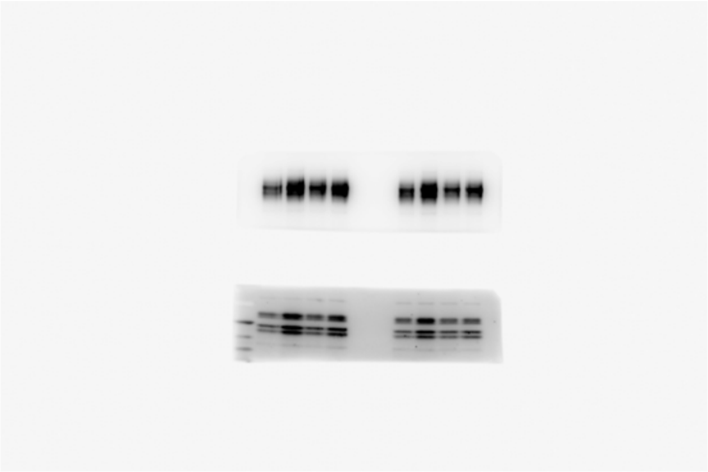

MAG+MBP

Fig. 5g

Hes-5

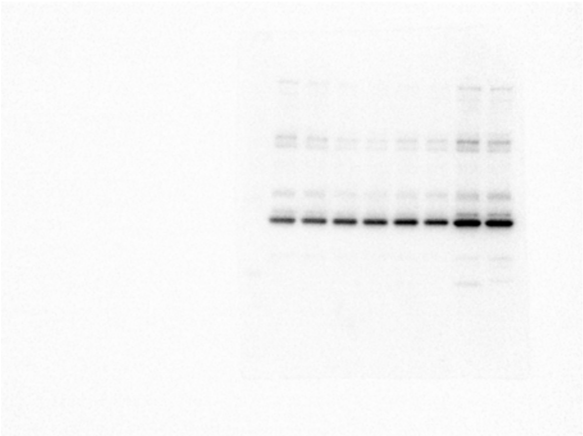

Actin

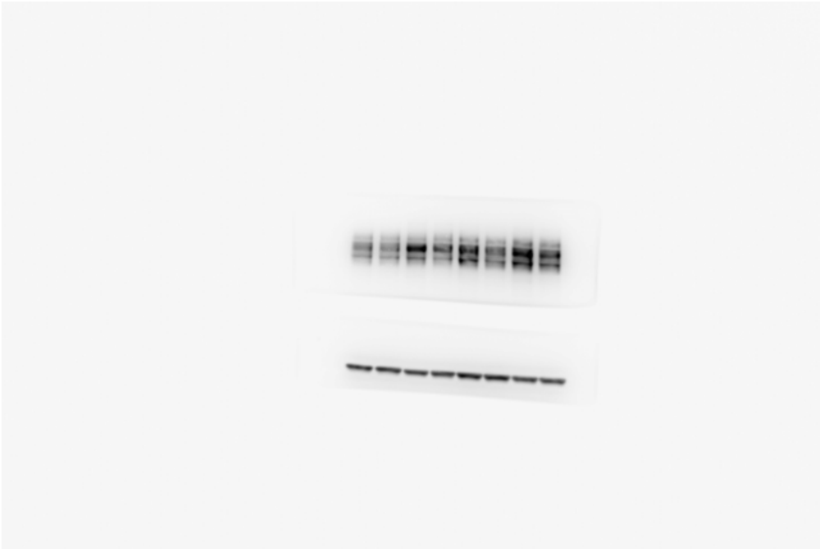

MAG+Actin

MBP

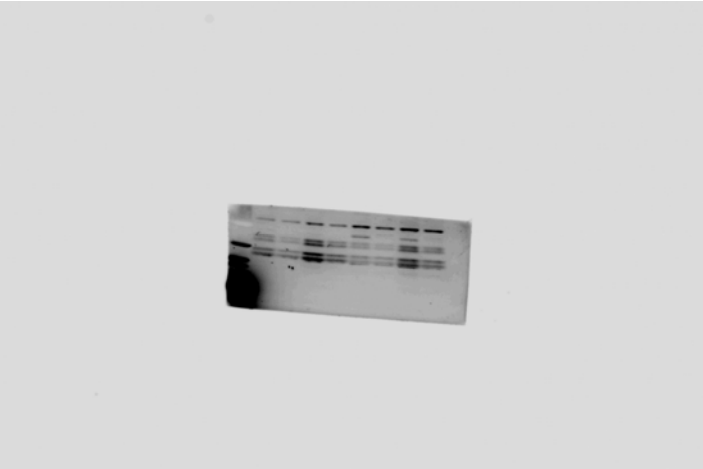

Supplement: S2 Fig — (PDF) [file pone.0189380.s002.pdf]
